# Cervical sagittal alignment changes following anterior cervical discectomy and fusion, laminectomy with fusion, and laminoplasty for multisegmental cervical spondylotic myelopathy

**DOI:** 10.1186/s13018-023-03640-9

**Published:** 2023-03-11

**Authors:** Xiang-Yu Li, Yu Wang, Wei-Guo Zhu, Cheng-Xin Liu, Chao Kong, Shi-Bao Lu

**Affiliations:** 1 Department of Orthopedics, Xuanwu Hospital, Capital Medical University, No.45 Changchun Street, Xicheng District, Beijing, China; 2 National Clinical Research Center for Geriatric Diseases, Beijing, China

**Keywords:** Cervical spondylotic myelopathy, Cervical sagittal alignment, Anterior cervical discectomy and fusion, Laminectomy and fusion, Laminoplasty

## Abstract

**Objective:**

Cervical sagittal alignment changes (CSACs) influence outcomes and health-related quality-of-life. Anterior cervical discectomy and fusion (ACDF), laminectomy with fusion (LCF), and laminoplasty (LP) are common treatments for multisegmental cervical spondylotic myelopathy; however, these approaches need to be compared.

**Methods:**

Our study included 167 patients who underwent ACDF, LCF, or LP. Patients were divided into four groups according to C2-C7 Cobb angle (CL): kyphosis (CL < 0°), straight (0° ≤ CL < 10°), lordosis (10° ≤ CL < 20°), and extreme lordosis (20° ≤ CL) groups. CSACs consist of two parts. CSAC from the preoperative period to the postoperative period is surgical correction change (SCC). CSAC from the postoperative period to the final follow-up period is postoperative lordosis preserving (PLP). Outcomes were evaluated using the Japanese Orthopaedic Association score and the neck disability index.

**Results:**

ACDF, LCF, and LP had equivalent outcomes. ACDF had greater SCC than LCF and LP. During follow-up, lordosis decreased in the ACDF and LCF groups but increased in the LP group. For straight alignment, ACDF had greater CSAC and greater SCC than the LCF and LP groups but similar PLP. For lordosis alignment, ACDF and LP had positive PLP, and LCF had negative PLP. For extreme lordosis, ACDF, LP, and LCF had negative PLP; however, cervical lordosis in the LP group was relatively stable during follow-up.

**Conclusions:**

ACDF, LCF, and LP have different CSAC, SCC, and PLP according to a four-type cervical sagittal alignment classification. Preoperative cervical alignment is an important consideration in deciding the type of surgical treatment in CSM.

**Supplementary Information:**

The online version contains supplementary material available at 10.1186/s13018-023-03640-9.

## Introduction

Cervical spondylotic myelopathy (CSM) is a common degenerative disease in elderly patients that leads to cervical spinal cord function impairment. To decompress the spinal cord and achieve good cervical alignment, surgery should be considered for patients who are refractory to conservative treatment.

The optimal surgical strategy for CSM remains controversial [1, 2]. Regardless of the surgical method, adequate decompression, reconstruction of the spine shape, and restoring stability are essential goals for CSM patients. Good cervical spine alignment is the bedrock of good outcomes [3].

Studies found that cervical and segmental lordosis improvements after the anterior approach were greater than those following the posterior approach [4, 5]. Liang et al. found that preservation of lordosis after anterior cervical discectomy and fusion (ACDF) was poor in patients with high cervical lordosis, preservation of lordosis after laminoplasty (LP) was good in patients with high cervical lordosis. ACDF is recommended only for patients with a cervical curvature index less than 20° [6]. Kong et al. found that laminectomy with fusion (LCF) corrected lordosis in patients with a low ratio of C2-C7 Cobb angle to T1 slope more than those with fair or high ratios [7]. Kuo et al. found that patients with high T1 slope had decreased cervical lordosis after cervical disc arthroplasty, whereas patients with low T1 slope had increased cervical lordosis after [8].

Several approaches to varying cervical sagittal alignments result in different cervical sagittal alignment changes (CSACs). In our opinion, there are two parts of CSAC from the preoperative period to the follow-up period. CSAC from the preoperative period to the postoperative period is surgical correction change (SCC), which reflects surgical correction. CSAC from the postoperative period to the final follow-up period is postoperative lordosis preserving (PLP), which reflects postoperative cervical curvature change. This study compared CSAC, SCC, and PLP after the anterior approach, LCF, or LP.

## Methods

### Patients

This study was approved by the institutional review board of the authors’ affiliated institutions. We retrospectively reviewed 167 consecutive patients who underwent ACDF, LCF, or LP from January 2016 to December 2019. Patients were eligible if they met the following inclusion criteria: (1) age 18 years or older; (2) cervical cord compression of three levels or more on imaging; (3) at least one clinical sign of myelopathy; and (4) at least 6 months of follow-up. Exclusion criteria included cervical instability, spondylolisthesis, cervical infection, ankylosis, malignancy, neurological disorder, post-traumatic myelopathy, and history of cervical spine surgery. Age, sex, decompressed levels, and follow-up period were reviewed and statistically analyzed.

### Surgical procedures

After the induction of general endotracheal anesthesia, patients were placed supine with extra cervical extension and underwent ACDF using the Smith-Robinson approach. The right side was chosen as the regular incision side. Discectomies were performed using microscopy. Poly-ether-ether ketone cages filled with allograft were inserted into the intervertebral spaces. Titanium plates and screws were used to fix the vertebral bodies.

In the LCF and LP groups, patients were positioned prone. An incision in the back of the neck was performed. The paraspinal muscles of patients were stripped away to expose the lamina. Patients in the LCF group underwent laminectomy followed by posterior instrumentation with lateral mass screws. Local bone autograft from the laminectomy was packed beneath and around the instrumentation. The open-door type of cervical en-bloc laminoplasty was performed for decompression in the LP group. One side of the lamina was opened, and the other side served as the hinge. Titanium miniplates or suture anchors were used to maintain the spinal canal space open.

### Imaging

**Fig. 1:**
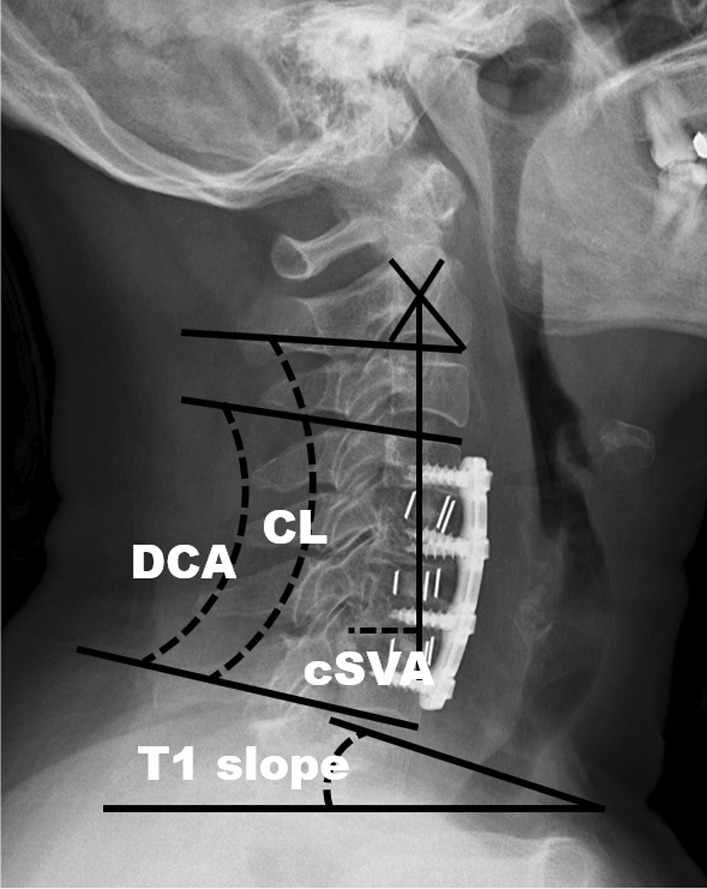
Visual representation of the measurement of CL, DCA, T1 slope, and cSVA. *CL, C2-C7* Cobb angle, *cSVA* C2-C7 sagittal vertical axis, *DCA* Decompressed Cobb angle

Cervical sagittal alignment parameters were measured before surgery, 1 week after surgery, and at the final follow-up with neutral standing lateral X-rays (Fig. 1). C2-C7 Cobb angle (CL) was defined as the C2 vertebra lower endplate angle and the C7 vertebra lower endplate. Decompressed Cobb angle (DCA) was defined as the Cobb angle between the inferior endplate of the superior vertebral body of decompressed levels and the inferior endplate of the caudal vertebral body of decompressed levels. The cSVA was defined as the distance from the posterosuperior corner of the C7 vertebral body and the vertical line from the center of the C2 vertebral body. T1 slope was defined as the angle between the horizontal plane and the line parallel to the superior T1 endplate. All data were measured and calculated by two spine surgeons.

The preoperative cervical alignment was classified into one of four types according to CL: kyphosis (CL < 0°), straight (0° ≤ CL < 10°), lordosis (10° ≤ CL < 20°), and extreme lordosis (20° ≤ CL). CSAC = (final CL) – (preoperative CL). SCC = (postoperative CL) – (preoperative CL). PLP = (final CL) – (postoperative CL).

### Clinical evaluation

The Japanese Orthopaedic Association (JOA) score was assessed according to symptoms. The recovery rate was calculated based on the following formula: recovery rate (%) = (postoperative JOA – preoperative JOA)/(total score – preoperative JOA) × 100% [9]. We administered the neck disability index (NDI), a self-assessment health-related quality of life questionnaire.

### Statistical analysis

All collected data were analyzed using IBM SPSS Statistics, version 22.0. (IBM Corp., Armonk, NY, USA). Statistical analysis was performed using the Kruskal–Wallis H test and one-way analysis of variance. A probability (p) value of ≤ 0.05 was considered statistically significant. Results were expressed as the mean value ± standard deviation.

## Results

A total of 167 patients were enrolled; 45 patients received ACDF, 55 patients received LCF, and 67 patients received LP. The mean postoperative follow-up period was 18.96 ± 6.85 months. Although ACDF had higher preoperative JOA scores (*p* = 0.041), the three surgical groups had similar clinical outcomes (JOA: *P* = 0.509, NDI: 0.960). The age of the LCF group was older than that of the ACDF and LP groups (*p* = 0.009). The LCF group had more operated levels than the other groups (*p* < 0.001). The three surgical groups' preoperative sagittal alignment parameters (CL, T1 slope, cSVA, and DCA) were similar. However, the postoperative and final sagittal alignment parameters (CL, T1 slope, and DCA) were more significant in the ACDF group than in the LCF or LP groups. Postoperative cSVA and final cSVA were lower in the ACDF group than in the LCF and LP groups (Table 1).

**Table 1:** Clinical and radiological characteristics of three surgical groups

	ACDF (45)	LCF (55)	LP (67)	*p*
Age (years)	59.18 ± 10.48	63.38 ± 9.71^#^	58.03 ± 9.11	0.009
Gender (M/F)	23/22	35/20	49/18	0.059
Follow-up period (months)	18.56 ± 7.73	19.29 ± 6.62	18.96 ± 6.50	0.870
*JOA score*				
Preoperative JOA score	11.40 ± 1.70*	10.56 ± 1.65	10.74 ± 1.74	0.041
Postoperative JOA score	14.87 ± 1.90	14.29 ± 1.98	14.31 ± 2.11	0.278
Recovery rate (%)	66.11 ± 23.71	61.24 ± 22.94	61.00 ± 26.54	0.509
*NDI*				
Preoperative NDI (%)	20.38 ± 17.02	21.96 ± 19.32	22.33 ± 19.32	0.856
Follow-up NDI (%)	6.58 ± 11.78	6.49 ± 11.25	7.13 ± 15.94	0.960
*CL*				
Preoperative CL (°)	14.35 ± 12.09	15.72 ± 9.63	18.44 ± 10.55	0.120
Postoperative CL (°)	20.29 ± 9.46*	11.72 ± 10.39	10.45 ± 10.70	< 0.001
Final CL (°)	19.49 ± 8.73*	10.92 ± 9.27	10.92 ± 9.32	< 0.001
*T1 slope*				
Preoperative T1 slope (°)	25.66 ± 8.65	24.88 ± 7.83	24.06 ± 7.24	0.567
Postoperative T1 slope (°)	28.42 ± 7.28*	24.92 ± 8.01	23.07 ± 7.20	0.001
Final T1 slope (°)	26.87 ± 7.10*	23.03 ± 7.63	21.56 ± 6.31	0.001
*cSVA*				
Preoperative cSVA (mm)	15.49 ± 11.10	20.58 ± 10.92	16.88 ± 12.45	0.072
Postoperative cSVA (mm)	16.93 ± 11.55*	27.84 ± 13.88	29.10 ± 16.59	< 0.001
Final cSVA (mm)	13.55 ± 8.31*	26.60 ± 13.68	25.88 ± 14.06	< 0.001
DCA				
Preoperative DCA (°)	11.97 ± 10.79	8.18 ± 9.26	9.10 ± 8.35	0.117
Postoperative DCA (°)	18.91 ± 9.12*	5.54 ± 9.80	6.44 ± 8.51	< 0.001
Final DCA (°)	16.70 ± 9.96*	4.26 ± 8.59	6.04 ± 8.13	< 0.001
*Decompression level*				
Numbers of levels operated	3.31 ± 0.51	4.01 ± 0.80^#^	3.51 ± 0.66	< 0.001

^*^The results in group ACDF had a significant difference from those in group LCF or LP

^#^The results in group LCF had a significant difference from those in group ACDF or LP

^%^The results in group LP significantly differed from that in group ACDF

*ACDF* Anterior cervical discectomy and fusion, *LCF* Laminectomy with fusion, *LP* Laminoplasty, *CL* C2-C7 Cobb angle, *cSVA* C2-C7 sagittal vertical axis, *DCA* Decompressed Cobb angle, *JOA* Japanese Orthopaedic Association, *NDI* Neck disability index

CSAC, SCC, and PLP of three surgical groups are presented in Table 2. After surgery, the lordosis angle improved significantly in the ACDF group but decreased in the LCF and LP groups. ACDF had greater SCC than LCF and LP (*P* < 0.001). Lordosis decreased during the follow-up period in the ACDF and LCF groups but increased in the LP group. The LP group had positive PLP, although there were no significant differences among the three groups (*P* = 0.196).

**Table 2:** CSAC, SCC, and PLP of three surgical groups

Characteristics	ACDF (47)	LCF (72)	LP (69)	*p*
CSAC (°)	5.14 ± 11.25^*^	− 5.37 ± 9.28	− 7.52 ± 9.61	< 0.001
SCC (°)	5.93 ± 8.66^*^	− 4.00 ± 9.19^#^	− 7.99 ± 10.00^%^	< 0.001
PLP (°)	− 0.79 ± 8.05	− 1.37 ± 4.44	0.47 ± 4.77	0.196

^*^The results in group ACDF had a significant difference from those in group LCF or LP

^#^The results in group LCF had a significant difference from those in group ACDF or LP

^%^The results in the LP group had a significant difference from the LCF and ACDF groups

*CSAC* Cervical sagittal alignment changes, *SCC* Surgical correction change, *PLP* Postoperative lordosis preserving, *ACDF* Anterior cervical discectomy and fusion, *LCF* Laminectomy with fusion, *LP* Laminoplasty

CSAC, SCC, and PLP of ACDF in different cervical alignments are shown in Table 3. Patients with larger cervical lordosis angle had less SCC (*p* < 0.001) and less CSAC (*p* < 0.001). Even in the extreme lordosis group, patients sustained lordosis loss after ACDF (*p* = 0.021).

**Table 3:** CSAC, SCC, and PLP of ACDF in different cervical alignments

	Kyphosis (5)	Straight (11)	Lordosis (14)	Extreme lordosis (15)	*p*
CSAC (°)	17.18 ± 13.10^#&^	13.07 ± 6.71*$	5.41 ± 8.52^^$&^	− 4.9 ± 6.22^*#^^	< 0.001
SCC (°)	14.66 ± 6.66^#^^	11.87 ± 7.08^*$^	3.47 ± 8.40^$^^	0.97 ± 5.84^*#^	< 0.001
PLP (°)	2.52 ± 10.14^#^	1.20 ± 7.09^^^	1.95 ± 5.32^*^	− 5.92 ± 8.32^*^#^	0.021

*CSAC* Cervical sagittal alignment changes, *SCC* Surgical correction change, *PLP* Postoperative lordosis preserving, *ACDF* Anterior cervical discectomy and fusion

^*, #, ^^ *P*-value of post hoc test < 0.05

CSAC, SCC, and PLP of LCF in different cervical alignments are displayed in Table 4. Patients with kyphosis or straight cervical alignment gained lordosis and positive SCC after LCF. However, patients with lordosis or extreme lordosis cervical alignment had negative SCC. Only kyphosis patients had positive PLP.

**Table 4:** CSAC, SCC, and PLP of LCF in different cervical alignments

	Kyphosis (3)	Straight (10)	Lordosis (23)	Extreme lordosis (19)	*p*
CSAC (°)	4.42 ± 5.69^#^	1.02 ± 8.47^*^^	− 5.50 ± 8.86^^^	− 10.13 ± 7.77^*#^	0.003
SCC (°)	2.94 ± 4.94	1.85 ± 8.76^*^	− 4.67 ± 8.38	− 7.36 ± 9.32^*^	0.033
PLP (°)	1.47 ± 2.15	− 0.83 ± 0.76	− 0.83 ± 4.90	− 2.76 ± 4.99	0.313

*CSAC* Cervical sagittal alignment changes, *SCC* Surgical correction change, *PLP* Postoperative lordosis preserving, *LCF* Laminectomy with fusion

^*, #, ^^ *P*-value of post hoc test < 0.05

CSAC, SCC, and PLP of LP in different cervical alignments are shown in Table 5. Only one patient with kyphosis cervical alignment underwent LP. Thus, we compared CSAC, SCC, and PLP among straight, lordosis, and extreme lordosis cervical alignment. LP had negative SCC, especially in the extreme lordosis group. However, patients with lordosis cervical alignment had positive PLP.

**Table 5:** CSAC, SCC, and PLP of LP in different cervical alignments

	Kyphosis (1)	Straight (17)	Lordosis (16)	Extreme lordosis (33)	pa
CSAC (°)	− 5.70	− 3.12 ± 7.11^*^	− 2.74 ± 7.35^^^	− 12.15 ± 9.79^*^^	< 0.001
SCC (°)	− 7.10	− 2.80 ± 7.97^*^	− 6.53 ± 7.10	− 11.14 ± 11.12^*^	0.011
PLP (°)	1.40	− 1.94 ± 4.47^^^	3.78 ± 3.79^*^^	− 0.75 ± 5.29^*^	0.005

A one-way analysis of variance was performed among straight, lordosis, and extreme lordosis group

*CSAC* Cervical sagittal alignment changes, *SCC* Surgical correction change, *PLP* Postoperative lordosis preserving, *LP* Laminoplasty

^*^, ^ *P*-value of post hoc test < 0.05

**Fig. 2:**
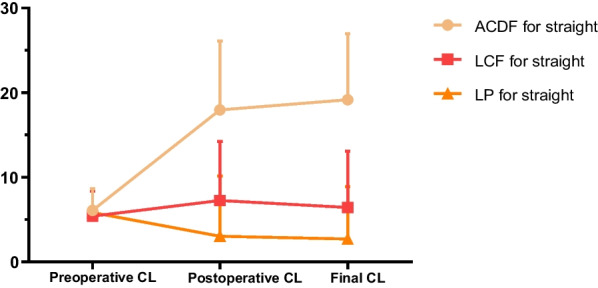
CL of ACDF, LCF, and LP in straight group at different time points (preoperative, postoperative, and final follow-up). *ACDF* Anterior cervical discectomy and fusion, *LCF* Laminectomy with fusion, *LP* Laminoplasty, *CL* C2-C7 Cobb angle

Figure 2 shows cervical lordosis change in three surgical groups for straight cervical alignment. Cervical lordosis angle changed larger after ACDF and LCF but decreased after LP for straight cervical alignment. Postoperative CL and final CL were larger in ACDF than in LCF and LP. When compared CSAC, SCC, and PLP of ACDF, LCF and LP in straight alignment, we found that ACDF had greater CSAC and SCC than in LCF and LP (*p* < 0.001, *p* < 0.001), but similar PLP (*p* = 0.528) (Table 6).

**Table 6:** CSAC, SCC, and PLP of ACDF, LCF, and LP in straight alignment

	ACDF (11)	LCF (10)	LP (17)	*p*
CSAC (°)	13.07 ± 6.71^*$^	1.02 ± 8.47^*^	− 3.12 ± 7.11^$^	< 0.001
SCC (°)	11.87 ± 7.08^*$^	1.85 ± 8.76^*^	− 2.80 ± 7.97^$^	< 0.001
PLP (°)	1.20 ± 7.09	− 0.83 ± 0.76	− 0.32 ± 3.08	0.528

^*, $^ *P*-value < 0.05

*CSAC* Cervical sagittal alignment changes, *SCC* Surgical correction change, *PLP* Postoperative lordosis preserving, *ACDF* Anterior cervical discectomy and fusion, *LCF* Laminectomy with fusion, *LP* Laminoplasty

**Fig. 3:**
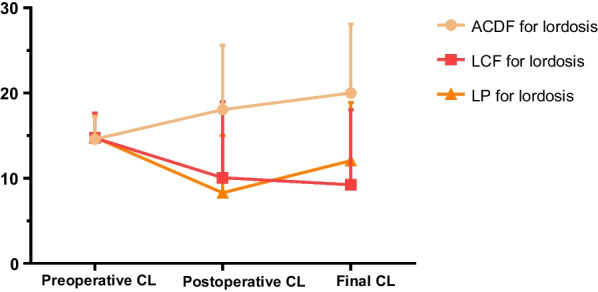
The CL value of ACDF, LCF, and LP in lordosis group at different times (preoperative, postoperative, and final follow-up). *ACDF* Anterior cervical discectomy and fusion, *LCF* Laminectomy with fusion, *LP* Laminoplasty, *CL* C2-C7 Cobb angle

Figure 3 shows cervical lordosis change in three surgical groups for lordosis cervical alignment. Only ACDF improved cervical lordosis angle after surgery. ACDF and LP had positive PLP. The cervical lordosis angle decreased after LCF for lordosis cervical alignment. CSAC and SCC were larger in ACDF than in LCF and LP (*p* = 0.001, *p* = 0.003). LP had greater PLP than in LCF (*p* = 0.0014) (Table 7).

**Table 7:** CSAC, SCC, and PLP of ACDF, LCF, and LP in lordosis alignment

	ACDF (14)	LCF (23)	LP (16)	*p*
CSAC (°)	5.41 ± 8.52^*$^	− 5.50 ± 8.86^*^	− 2.74 ± 7.35^$^	0.001
SCC (°)	3.47 ± 8.40^*$^	− 4.67 ± 8.38^*^	− 6.53 ± 7.10^$^	0.003
PLP (°)	1.95 ± 5.32	− 0.83 ± 4.90^*^	3.78 ± 3.78^*^	0.014

^*, $^ *P*-value < 0.05

*CSAC* Cervical sagittal alignment changes, *SCC* Surgical correction change, *PLP* Postoperative lordosis preserving, *ACDF* Anterior cervical discectomy and fusion, *LCF* Laminectomy with fusion, *LP* Laminoplasty

**Fig. 4:**
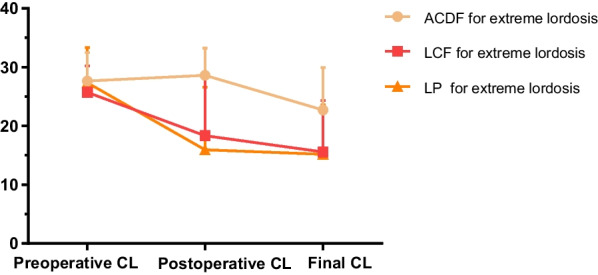
The CL value of ACDF, LCF, and LP in the extreme lordosis group at different time points (preoperative, postoperative, and final follow-up). *ACDF* Anterior cervical discectomy and fusion, *LCF* Laminectomy with fusion, *LP* Laminoplasty, *CL, C2-C7* Cobb angle

Figure 4 shows cervical lordosis change in three surgical groups for extreme lordosis cervical alignment. SCC was larger in ACDF than in LCF and LP. ACDF, LP, and LCF had negative CSAC. Cervical lordosis in the LP group was relatively stable during follow-up. LP had greater PLP than the ACDF (*p* = 0.027) (Table 8).

**Table 8:** CSAC, SCC, and PLP of ACDF, LCF, and LP in extreme lordosis alignment

	ACDF (15)	LCF (19)	LP (33)	*p*
CSAC (°)	− 4.9 ± 6.22^*^	− 10.13 ± 7.77	− 12.15 ± 9.79^*^	0.031
SCC (°)	0.97 ± 5.84^*$^	− 7.36 ± 9.33*	− 11.40 ± 11.12^$^	0.001
PLP (°)	− 5.92 ± 8.32^*^	− 2.77 ± 4.99	− 0.75 ± 5.29^*^	0.027

^*, $^ *P*-value < 0.05

*CSAC* Cervical sagittal alignment changes, *SCC* Surgical correction change, *PLP* Postoperative lordosis preserving, *ACDF* Anterior cervical discectomy and fusion; *LCF* Laminectomy with fusion, *LP* Laminoplasty

## Discussion

In this study, we found that ACDF, LCF, and LP have different CSAC, SCC, and PLP according to a four-type cervical sagittal alignment classification. The preoperative shape of cervical spine should be considered in the choice of operation. CSM is a common degenerative disease leading to cervical spinal cord function impairment. The treatment of CSM is based on retrodiscal or retro vertebral compression. The ACDF is used in retrodiscal compression, while laminectomy and fusion or laminoplasty is useful in retro vertebral compression. For patients with multi-level cervical canal stenosis, posterior cervical surgery is mainly considered, and fusion surgery is considered for patients with preoperative cervical kyphosis or instability to prevent the aggravation of cervical deformity. The aims of the surgical method are decompression of the spinal cord, restoration of cervical lordosis, and avoidance of kyphosis. Lordosis correction and preservation are essential for radiological outcomes. Good cervical spine alignment is the bedrock of good outcomes [3]. Cervical kyphosis or lordosis malalignment might negatively influence clinical outcomes [10, 11]. Studies found that ACDF improved cervical lordosis more than LP and LCF [12–14]. LP and LCF decreased lordosis after surgery [15]. ACDF and LCF correct lordosis because of fixation and fusion. LP has relatively good spinal flexibility. Thus, ACDF, LP, and LCF generate different CSACs after surgery.

Studies found that LP had superior clinical and radiological outcomes for cervical lordosis angle patients. For C2-C7 Cobb angle more than 20°, LP was associated with better pain outcomes than LCF [16]. For curvature index more than 20, LP had better lordosis preserving abilities than ACDF [6]. LP had relatively better PLP in the lordosis and extreme lordosis groups in the present study. However, LP had poor SCC and PLP in the straight group. LP demonstrated no fusion ability to maintain cervical alignment balance for cervical kyphosis patients. LP was not recommended for kyphosis deformity [10]. Studies found that T1 slope correlated with loss of lordosis after LP [17, 18]. We also found that loss of lordosis was significant in the extreme lordosis group after LP. There is a correlation between T1 slope and cervical lordosis angle [19, 20], which means that the extreme lordosis group demonstrated a relatively high T1 slope leading to a significant loss of lordosis. However, cervical alignment remained more stable during follow-up after LP than after ACDF in the extreme lordosis group. ACDF had a substantial loss of lordosis during follow-up in the extreme lordosis group. Longer-term follow-up is needed to compare postoperative cervical alignment after LP and ACDF for lordosis and extreme lordosis.

LCF and ACDF are fusion surgeries and can correct cervical alignment. The correction decreased with increased cervical lordosis in the LCF and ACDF groups. ACDF improved cervical lordosis in the kyphosis, straight, and lordosis groups in the present study. LCF increased cervical lordosis angle for kyphosis and straight alignment. ACDF had greater SCC than LCF, confirmed by Cabraja et al. [4]. Wang et al. found that cervical lordosis in the LCF and ACDF groups improved at 1 week, 3 months, 6 months, 12 months postoperatively but decreased at 18 and 24 months postoperatively [13]. Guo et al. found that three-level ACDF had a poor ability to maintain lordosis [21]. Studies found that cervical sagittal alignment change after ACDF was not associated with clinical outcomes [22, 23]. Thus, cervical alignment maintenance and restoration had limited effect on clinical outcomes. Adequate decompression remains fundamental to achieving good clinical outcomes. However, patients with severe kyphosis or sagittal imbalance have worse outcomes [10, 11, 24]. Thus, cervical sagittal alignment changes after surgery still need attention.

In the present study, ACDF provided poor PLP in the extreme lordosis group, and LCF had an excellent ability to maintain lordosis only in the kyphosis group. Only three ossification of the posterior longitudinal ligament (OPLL) patients in LCF group were included in this study. The range of motion of cervical vertebra may be limited in OPLL patients. We also found the cervical alignment correction and change after LCF. Nevertheless, the reasons for lordosis keeping loss after fusion surgery are unclear. Bony un-fusion, cage subsidence, loss of disc height, or adjacent pathology may be attributed to loss of cervical lordosis. A follow-up study is needed to identify the mechanisms of lordosis loss after fusion surgery.

Many surgeons proposed surgical decision strategies for CSM [25–27]. Because of the simplicity of the procedure, multi-level CSM with lordosis alignment was recommended for posterior decompression. One- to three-level CSM with kyphosis alignment should include anterior decompression because of its substantial correction ability. Anterior decompression is used widely for multi-level CSM. However, few studies compare correction and preservation abilities among ACDF, LCF, and LP for multi-level CSM. The results of this study could provide a basis for surgical decision-making. Because of the substantial correction ability, ACDF is recommended for any type of cervical alignment. LCF delivers fair correction ability and is recommended for kyphosis and straight cervical alignment. LP had no correction ability but good preserving ability for lordosis cervical alignment. ACDF decreased cervical lordosis during follow-up for extreme lordosis patients, but it was maintained in the LP group. LP was recommended for patients with cervical lordosis angle of more than 10°. Cases are shown in Additional file 1.

This study has several limitations owing to its retrospective design and selection bias. The difference in patient characteristics might have influenced the results. Because of a lack of patient cohorts, we analyzed three- to five-level decompression surgery together. The complex cervical deformity was not included because of mixed surgery requirements. The operative method was chosen according to its characteristics and the surgeon’s experience. Due to the limitations of retrospective studies, the details of the decision-making process were not collected; therefore, we are unable to comment on the process. We believed that the surgeons were unbiased in the decision-making process. The follow-up time is relatively short. More extended follow-up periods and larger patient cohorts are needed to confirm these findings. Because of the limited number of full-length X-rays, the overall sagittal balance was not considered and requires further study.

## Conclusion

ACDF, LCF, and LP have different CSAC, SCC, and PLP according to a four-type cervical sagittal alignment classification. The preoperative sagittal alignment guides surgical treatment based on the extent of cervical lordosis. LP is recommended for patients with cervical lordosis angle of more than 10°. LCF is recommended for patients with cervical lordosis angle less than 10°. ACDF is recommended for any type of cervical alignment.

## Supplementary Information

**Additional file 1**. Cases of three surgical methods.

## Data Availability

The datasets used and/or analysed during the current study are available from the corresponding author upon reasonable request.
