# Supplementary material for: Cervical sagittal alignment changes following anterior cervical discectomy and fusion, laminectomy with fusion, and laminoplasty for multisegmental cervical spondylotic myelopathy
Source: J Orthop Surg Res. 2023 Mar 11;18:190. doi: 10.1186/s13018-023-03640-9 (PMC10007737; doi:10.1186/s13018-023-03640-9)
Supplement: Supplementary file 1 — Additional file 1. Cases of three surgical methods. [file 13018_2023_3640_MOESM1_ESM.docx]

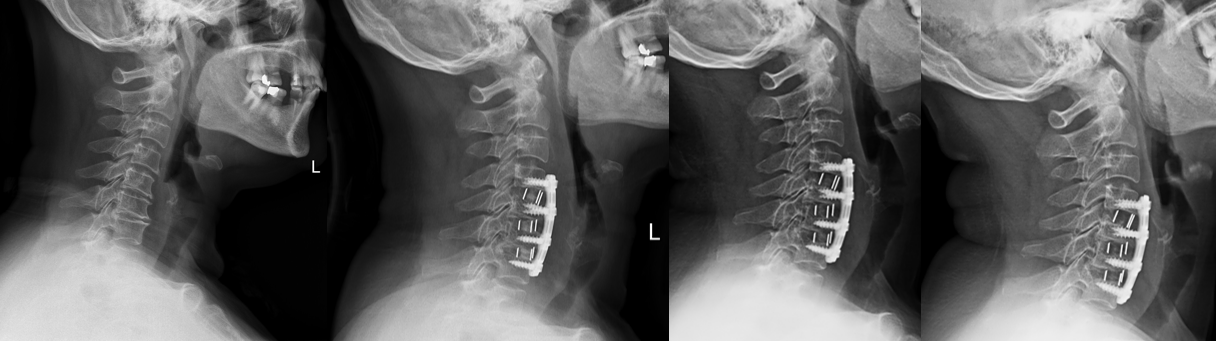


A 61-year-old female with kyphosis cervical alignment underwent ACDF. Lordosis change was 23.3°. Lordosis correction was 18.4°. lordosis preserving was 4.9°. Cervical lordosis angle increased after surgery.


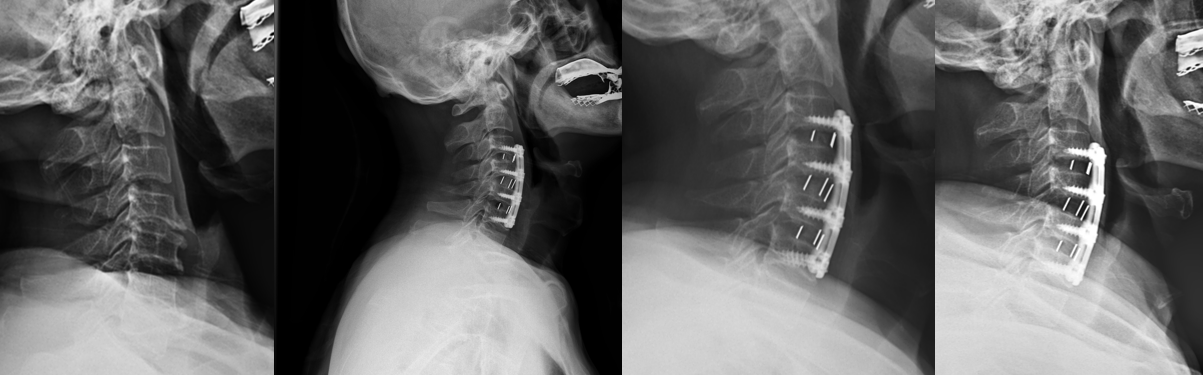


A 62-year-old male with extremely lordosis cervical alignment received ACDF. Lordosis change was -9.7°. Lordosis correction was -4.7°. lordosis preserving was -5.0°. Cervical lordosis angle kept decreasing after surgery.


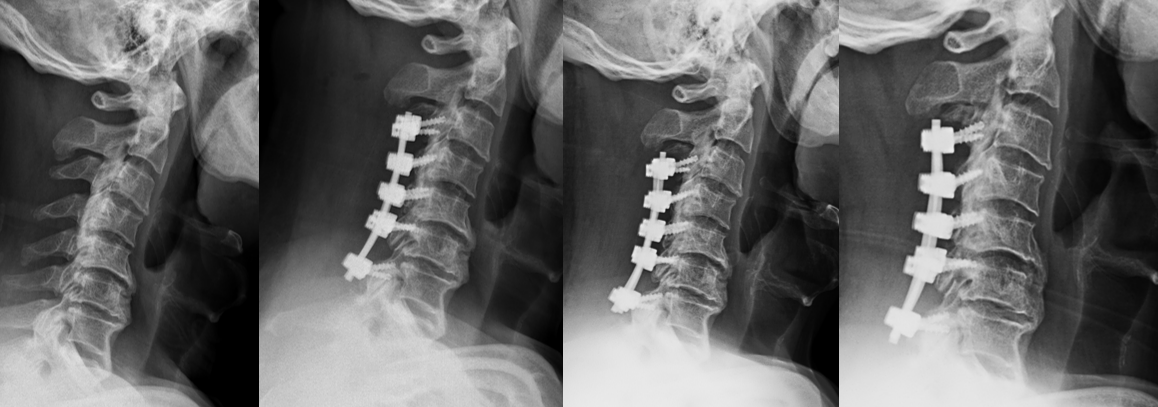


A 68-year-old male with kyphosis cervical alignment received LCF. Lordosis change was 9.7°. Lordosis correction was 7.6°. lordosis preserving was 2.1°. Cervical angle increased gradually after surgery.


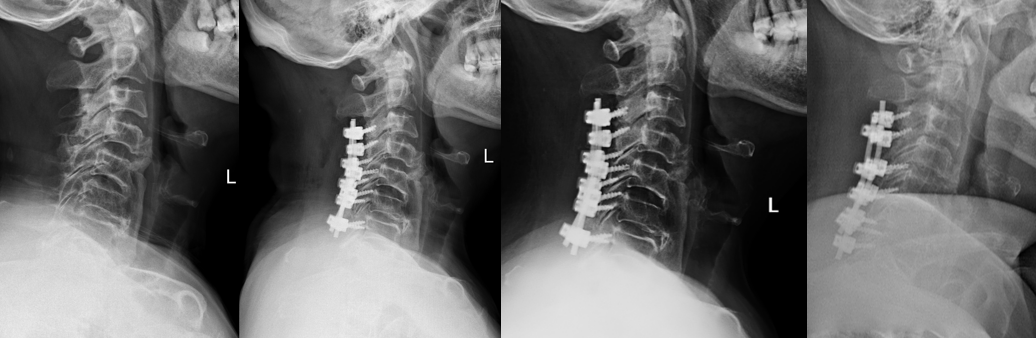


A 65-year-old female with kyphosis cervical alignment received LCF. Lordosis change was -11.1°. Lordosis correction was -5.9°. lordosis preserving was -5.2°. Cervical angle decreased gradually after surgery.


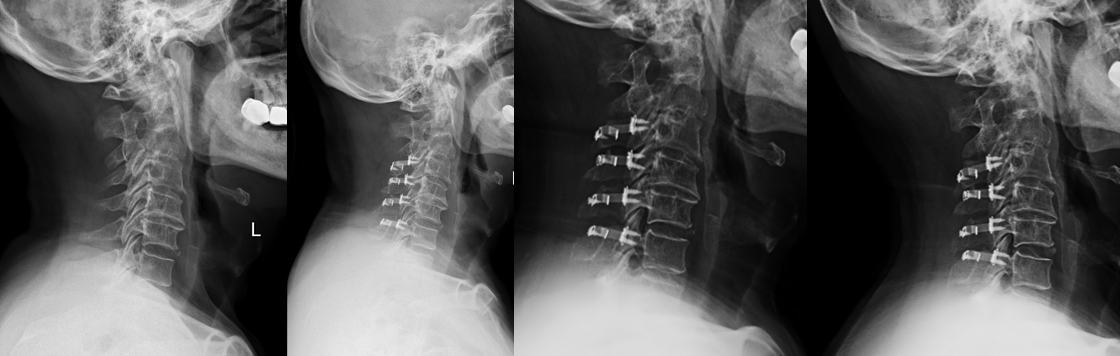


A 62-year-old male with lordosis cervical alignment received LP. Lordosis change was -0.6°. Lordosis correction was -14.1°. lordosis preserving was 13.5°. Cervical angle decreased immediately after surgery and increased during follow-up.


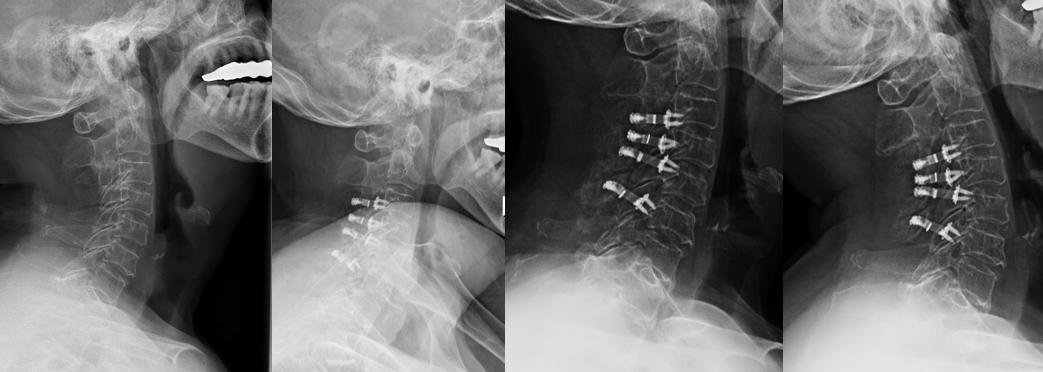


A 71-year-old female with extremely lordosis cervical alignment received LP. Lordosis change was 4.6°. Lordosis correction was -9.2°. lordosis preserving was 13.8°. Cervical angle decreased immediately after surgery and increased during follow-up.
